# Next-Generation Biomaterials for Wound Healing: Development and Evaluation of Collagen Scaffolds Functionalized with a Heparan Sulfate Mimic and Fibroblast Growth Factor 2

**DOI:** 10.3390/jfb16020051

**Published:** 2025-02-07

**Authors:** Merel Gansevoort, Sabine Wentholt, Gaia Li Vecchi, Marjolein de Vries, Elly M. M. Versteeg, Bouke K. H. L. Boekema, Agnes Choppin, Denis Barritault, Franck Chiappini, Toin H. van Kuppevelt, Willeke F. Daamen

**Affiliations:** 1 Department of Medical BioSciences, Research Institute for Medical Innovation, Radboud University Medical Center, 6525 GA Nijmegen, The Netherlands; 2 Burn Research Lab, Alliance of Dutch Burn Care, 1941 AJ Beverwijk, The Netherlands; 3 Department of Plastic, Reconstructive and Hand Surgery, Amsterdam UMC Location Vrije Universiteit Amsterdam, 1081 HV Amsterdam, The Netherlands; 4 OTR3, 75001 Paris, France

**Keywords:** biomaterial, wound healing, skin regeneration, collagen, heparin/heparan sulfate, fibrosis

## Abstract

Fibrosis after full-thickness wound healing—especially after severe burn wounds—remains a clinically relevant problem. Biomaterials that mimic the lost dermal extracellular matrix have shown promise but cannot completely prevent scar formation. We present a novel approach where porous type I collagen scaffolds were covalently functionalized with ReGeneRating Agent (RGTA^®^) OTR4120. RGTA^®^ is a glycanase-resistant heparan sulfate mimetic that promotes regeneration when applied topically to chronic wounds. OTR4120 is able to capture fibroblast growth factor 2 (FGF-2), a heparan/heparin-binding growth factor that inhibits the activity of fibrosis-driving myofibroblasts. Scaffolds with various concentrations and distributions of OTR4120 were produced. When loaded with FGF-2, collagen–OTR4120 scaffolds demonstrated sustained release of FGF-2 compared to collagen–heparin scaffolds. Their anti-fibrotic potential was investigated in vitro by seeding primary human dermal fibroblasts on the scaffolds followed by stimulation with transforming growth factor β1 (TGF-β1) to induce myofibroblast differentiation. Collagen–OTR4120(-FGF-2) scaffolds diminished the gene expression levels of several myofibroblast markers. In absence of FGF-2 the collagen–OTR4120 scaffolds displayed an inherent anti-fibrotic effect, as the expression of two fibrotic markers (TGF-β1 and type I collagen) was diminished. This work highlights the potential of collagen–OTR4120 scaffolds as biomaterials to improve skin wound healing.

## 1. Introduction

Scar formation that follows the destruction of the epidermis and dermis in third-degree burns remains a severe complication for many patients [1]. Such full-thickness wounds are unable to heal without clinical intervention and, in most cases, this entails the use of split-thickness skin transplants [2]. Although this approach ensures fast re-epithelialization of the wound area, the formation of scar tissue remains a problem as dermal restoration is far from optimal in many cases [3]. Skin substitutes that act as a (temporary) dermal layer can improve wound healing efficiently and limit the fibrotic response through the incorporation of anti-fibrotic molecules [4,5].

Fibrosis is generally described as an overzealous wound healing response. Of the different wound healing phases—hemostasis, inflammation, proliferation, and remodeling—the proliferation phase has a strong impact on scar formation. During this phase, fibroblasts migrate to the wound site and differentiate into myofibroblasts under the influence of changing tissue mechanical characteristics and the presence of transforming growth factor beta 1 (TGF-β1) [6]. Active myofibroblasts are characterized by the incorporation of α-smooth muscle actin (α-SMA) stress fibers in the cytoskeleton, allowing them to contract the extracellular matrix (ECM) [7]. In addition, active myofibroblasts deposit large amounts of ECM. Matrix contraction and ECM deposition increase matrix stiffness, stimulating the mechanosensitive myofibroblasts to contract and in turn releasing more TGF-β1 from its latency-associated peptide into the environment [8]. The result is a positive feedback loop that drives scar formation.

Diminishing myofibroblast activity reduces fibrosis [9]. In wound healing, the combination of a three-dimensional scaffold component together with (anti-fibrotic) effector molecules that support tissue regeneration has shown potential in vivo [10,11,12]. Type I collagen is an attractive scaffold component due to its excellent biocompatibility and biodegradability [13,14]. Moreover, type I collagen is the most abundant ECM protein in skin, making it an excellent choice for skin substitutes [15,16]. Fibroblast growth factor 2 (FGF-2) is a potent myofibroblast differentiation inhibitor, as it prevents the TGF-β-mediated fibroblast-to-myofibroblast transition [17]. In the natural ECM, FGF-2 is captured and presented by heparan sulfates (HSs) via its heparin-binding domains. HSs control the interaction between FGF-2 and its receptors, thereby controlling FGF-2 signaling [18,19]. HSs and structurally similar—but more sulfated—heparin are both glycosaminoglycans (GAGs) and have been used to capture FGF-2 on collagenous biomaterials [20,21]. This approach is promising for wound healing applications, and further development of such scaffolds will enhance their functionality and maximize their chances for clinical success.

One considerable drawback related to this approach is the highly degradative nature of the wound environment, which leads to the rapid breakdown of collagens, HS, and growth factors, such as FGF-2, when the protection of HS is removed [22,23]. A solution was to reconstruct the ECM in the form of heparan sulfate mimetics, known as a ReGeneraTing Agent (RGTA^®^, OTR3, Paris, France). These dextran-based synthetic polymers contain α1-6 glycosidic bonds between subunits, which are not recognized by endogenous glycosidases and make them more resistant to enzymatic degradation [24]. Moreover, RGTA^®^ mimics the function of heparan sulfates in its ability to bind ECM molecules and heparin-binding growth factors, such as FGF-2. A clinically approved RGTA^®^, OTR4120, in a medical device (CACIPLIQ20^®^, OTR3, Paris, France) has demonstrated its pro-regenerative effects in chronic wounds by stabilizing the wound environment and improving wound healing [25]. So far, the use of RGTA^®^ in wound healing has been limited to topical applications with creams or water-based formulations as a spray or solution (CACIPLIQ20^®^).

We present a novel approach where collagen scaffolds are functionalized with the RGTA^®^ OTR4120 to capture and protect FGF-2. The combination of a porous collagen scaffold that acts as a dermal replacement and the stabilizing influence of OTR4120 and anti-fibrotic effect of FGF-2 may improve the performance of biomaterials in wound healing. OTR4120 covalently crosslinked to type I collagen fibrils is an approach that has not yet been investigated and could offer new administration routes for RGTA^®^ in addition to existing solution-based approaches. In this study, we present the scaffold production process and demonstrate that OTR4120 retains its growth factor-protecting characteristics, even when chemically crosslinked to the biomaterial. Lastly, we demonstrate the ability of these OTR4120-functionalized collagen scaffolds to limit the fibrotic response in vitro.

## 2. Materials and Methods

### 2.1. Production of Scaffolds

Porous collagen scaffolds were prepared from purified bovine type I collagen fibrils [21]. The fibrils were suspended in 0.25 M acetic acid (Sigma-Aldrich, Saint Louis, MO, USA) to a final concentration of 0.8% *w*/*v* and swollen overnight at 4 °C under constant agitation. The resulting suspension was homogenized on ice using a Potter–Elvehjem glass tube with a pestle (0.35 mm intervening space, Louwers Glass and Ceramic technologies, Hapert, The Netherlands). Air bubbles were removed by centrifugation at 525 g for 30 min at 4 °C. Porous scaffolds were created by pouring the collagen suspension into molds (6-well cell culture suspension plates, 4 mL per well, Greiner Bio-One, Alphen aan den Rijn, The Netherlands) and freezing it for 4 h at −20 °C, followed by lyophilization for 4.5 h at −20 °C, 4 h at 0 °C, and 1.5 days at 20 °C with pressure < 80 mTorr and temperature increases of 0.17 °C/min (LP-03 table-top R&D freeze dryer, ilShin BioBase, Ede, The Netherlands). Two methods were tested to add OTR4120 (OTR3, Paris, France) or heparin (Organon, Oss, The Netherlands) to the scaffolds. In the “mixed” method, heparin or OTR4120 was added to the acetic acid at the start of the swelling phase. In the “soaked” method, the compounds were added to the dry porous scaffolds before the chemical crosslinking. OTR4120 or heparin was added at a final concentration of 0.05% (0.5 mg/mL collagen suspension), 0.025%, 0.0125%, or 0.00625%, with “no addition” (0%) as a control.

Scaffolds belonging to the “mixed” condition were immediately crosslinked for 3 h under constant agitation using 33 mM 1-ethyl-3-(3-dimethyl aminopropyl) carbodiimide (EDC, Sigma-Aldrich) and 6 mM N-hydroxysuccinimide (NHS, Bachem AG, Bubendorf, Switzerland) in MES buffer (50 mM 2-morpholinoethane sulphonic acid (MES, Sigma-Aldrich) with 40% ethanol, pH 5.0). Scaffolds from the “soaked” condition were first incubated overnight in half the volume of MES buffer supplemented with 2× concentrated OTR4120 or heparin under constant agitation. Thereafter, “soaked” scaffolds were crosslinked for 3 h under constant agitation by adding the same volume of 66 mM EDC/12 mM NHS in MES buffer. After crosslinking, both “mixed” and “soaked” scaffolds were washed in 0.1 M Na_2_HPO_4_ (Sigma-Aldrich), 1 M NaCl (Sigma-Aldrich), 2 M NaCl (Sigma-Aldrich), and demineralized water, frozen at −20 °C, and dried by lyophilization. Scaffolds were sterilized by gamma irradiation using a total dose of 32–36 kGy (in accordance with EN ISO 111371-1 [26] and EN ISO 13485 [27]).

Scaffolds were incubated with 3.5 µg/mL of human recombinant fibroblast growth factor 2 (FGF-2, 146 amino acids, animal-free, PeproTech^®^, Thermo Scientific, Waltham, MA, USA) in phosphate-buffered saline (PBS, 684 mM NaCl, 13 mM g KCl, 49 mM, Na_2_HPO_4_, 9 mM KH_2_PO_4_ in H_2_O, pH 7.2, all chemicals from Sigma-Aldrich) for 2.5 days under constant agitation. Scaffolds were washed 3 × 15 min in PBS to remove unbound FGF-2 and processed for further analysis.

### 2.2. Scaffold Characterization

#### 2.2.1. Quantification of OTR4120 and Heparin Content

The OTR4120/heparin content in the scaffolds was measured via Alcian blue staining using dot blots [28]. Collagen scaffolds were digested in triplicate at 65 °C in 2.5 U/mL papain (from Papaya latex, Sigma-Aldrich) in 50 mM NaPO_4_ (pH 6.5, Sigma-Aldrich) and spotted onto a positively charged PVDF membrane (0.45 µm, Thermo Scientific) along with standards of OTR4120 and heparin. Blots were stained with Alcian blue for 30 min: 2.5% *w*/*v* Alcian blue (Sigma-Aldrich, in 50% ethanol) diluted 10× in staining solution (0.4 M guanidium chloride (Sigma-Aldrich), 0.018 M H_2_SO_4_ (Sigma-Aldrich), and 0.25% *v*/*v* Triton-X-100, Sigma-Aldrich). The blots were then rinsed with demineralized water and the color intensity of the spots was measured using the Gel Doc XR+ System (Version 5.0, Bio-Rad, Hercules, CA, USA). The OTR4120/heparin content of the samples was calculated in µg OTR4120/heparin per mg collagen (*n* = 3).

#### 2.2.2. Effect of γ-Irradiation on Collagen Scaffolds

Samples from collagen scaffolds before and after sterilization with γ-irradiation (*n* = 3) were collected in reducing sample buffer (0.04% *w*/*v* bromophenol blue (Sigma-Aldrich), 1.0% *w*/*v* sodium dodecyl sulfate (SDS, Sigma-Aldrich), 1.25% *v*/*v* β-mercaptoethanol (Sigma-Aldrich, 2.5% *v*/*v* glycerol (Thermo Scientific, and 31.25 mM TRIS (Sigma-Aldrich) in Milli-Q water (pH 6.8)). Purified type I collagen fibrils were prepared simultaneously as a control. The samples were heated at 70 °C for 10 min and loaded onto 8% polyacrylamide gels (Thermo Scientific) together with PageRuler^TM^ Plus Prestained Protein Ladder (10–250 kDa, Thermo Scientific). Proteins were separated under reducing conditions and gels were washed briefly in demineralized water following completion of the run. Gels were heated in a microwave until boiling in 1% *w*/*v* Coomassie brilliant blue (MP Biomedicals, Eschwege, DE) dissolved in a staining solution of 10% glacial acetic acid (Sigma-Aldrich) and 50% methanol (Sigma-Aldrich) in demineralized water and left to stain for 15 min under constant agitation. The gels were washed twice for 20 min in the staining solution and then transferred to demineralized water for 30 min and photographed digitally.

#### 2.2.3. Localization of Heparin, OTR4120, and FGF-2

The distribution of OTR4120/heparin and FGF-2 on the scaffolds was visualized using indirect fluorescence assays on cryosections. Scaffolds were soaked in Tissue-Tek O.C.T. compound (Sakura Finetek, Alphen aan den Rijn, The Netherlands) for 20 min, frozen on dry ice, and stored at −80 °C. Cryosections of 7 µm were cut using a cryotome, mounted on Superfrost^TM^Plus Adhesion Microscope Slides (Epredia, Portsmouth, NH, USA), dried overnight, and stored at −20 °C until use. Before staining, sections were thawed and blocked in 2% *w*/*v* bovine serum albumin (BSA, fraction V, Roche, Basel, Switzerland) in PBS with 0.1% *v*/*v* Tween-20 (PBST, Sigma-Aldrich) for 30 min (BSA-PBST). OTR4120 and heparin were labeled with the single-chain antibody HS4C3-VSV (1:10), followed by incubation with mouse-anti-VSV P5D4 hybridoma supernatant (1:10) [29] and lastly incubation with goat-anti-mouse IgG (H+L) Alexa Fluor^TM^ 594 (1:500, Invitrogen, Thermo Scientific). FGF-2 was labeled with rabbit-anti-FGF-basic (1–24) (1:1000, Sigma-Aldrich) followed by incubation with goat-anti-rabbit (H+L) Alexa Fluor^TM^ 488 (1:500, Invitrogen). Each antibody was diluted in BSA-PBST and incubated on sections for 60 min. After every incubation step, the sections were washed 3 × 5 min with PBST. Finally, the sections were fixed 10 s in 97% ethanol (Sigma-Aldrich), mounted with Mowiol 4–88 reagent (Merck, Darmstadt, Germany), and enclosed with a cover glass. Sections were imaged using the ZOE^TM^ Fluorescent Cell Imager (Bio-Rad).

#### 2.2.4. Quantification of FGF-2 Captured by Scaffolds

Collagen scaffolds loaded with FGF-2 (*n* = 3) and FGF-2 solutions in PBS (for the standard curve) were stored at −20 °C in reducing sample buffer. Samples were boiled for 10 min, loaded onto 15% polyacrylamide gels (Thermo Scientific), and separated as described in Section 2.2.2, after which proteins were blotted onto nitrocellulose membranes (0.2 µm, Bio-Rad). Blots were washed once in PBS and blocked overnight at 4 °C in 2% *w*/*v* BSA-PBST under constant agitation, and FGF-2 was labeled for 60 min at room temperature with rabbit-anti-FGF-basic (1–24) (1:1000 in BSA-PBST, Invitrogen). Blots were washed three times for 5 min in PBST and then incubated for 60 min with goat-anti-rabbit IgG IRDye 800CW (1:15,000 in BSA-PBST, LI-COR, Lincoln, NE, USA). Following 3 × 5 min washes with PBST, the blots were scanned with the Odyssey^®^ CLx infrared imaging system (LI-COR) and the signal was quantified using Image Studio^TM^ Version 5.0 (LI-COR). The amount of FGF-2 captured was calculated in μg FGF-2/mg scaffold.

#### 2.2.5. Quantification of FGF-2 Release by Scaffolds

Gamma-sterilized collagen scaffolds were loaded with FGF-2 as described above and samples were transferred to Protein LoBind^®^ Tubes (Eppendorf, Thermo Scientific). The samples were incubated with 1 mL PBS supplemented with 100 U/mL penicillin and 100 μg/mL streptomycin (pen-strep, Gibco, Thermo Scientific) at 37 °C. PBS was replaced every few days (on days 1, 3, 5, 10, 15, 20, 25, and 30) and the incubation fluids were stored at −20 °C. The amount of FGF-2 released into the supernatants was quantified using a human FGF-2 ELISA kit (Invitrogen, Thermo Scientific) according to the manufacturer’s instructions. Data were processed as ng FGF-2/mg scaffold and presented as mean ± SD (*n* = 3; data were not tested for significant differences). After the final supernatant collection, the scaffolds were stored at −20 °C in reducing sample buffer. The amount of FGF-2 still bound on the scaffolds was determined via SDS-PAGE and Western blotting (Section 2.2.4); data were presented as mean ± SD (*n* = 3; data were not tested for significant differences).

### 2.3. Anti-Fibrotic Response In Vitro

#### 2.3.1. Cell Culture

Primary human dermal fibroblasts (HDFs, *n* = 3 donors) were provided by Dr. Boekema, cultured in Dulbecco’s Modified Eagle Medium (DMEM, Gibco) supplemented with 1× GlutaMax^TM^ (Gibco), pen-strep (Gibco), and 10% fetal bovine serum (FBS, HyClone^TM^, Cytiva Life Sciences, Marlborough, MA, USA), and kept in a culture incubator at 5% CO_2_, 37 °C, and atmospheric O_2_. HDFs were used in experiments before passage 5.

For cell culture experiments, Ø 12 mm scaffolds were punched out and sterilized using gamma irradiation (Section 2.1). All ensuing steps were carried out in aseptic conditions. Scaffolds were incubated with or without 3.5 µg FGF-2/mL PBS 1× for 2.5 days under constant agitation. Scaffolds were then washed 3 × 15 min in PBS to remove unbound FGF-2. Next, the scaffolds were placed on autoclaved Whatman paper to remove excess PBS and transferred to 12-well cell culture suspension plates (Greiner Bio-One); 3 × 10^5^ cells were seeded on top of each scaffold in a volume of 100 µL culture medium. Plates were placed in the culture incubator immediately after seeding and, after 3 h, the wells were filled with culture medium. Then, 21 h later, the medium was replaced with starvation medium consisting of DMEM, 1× GlutaMAX^TM^, pen-strep, and 0.5% FBS. Following 24 h of acclimatization to low-serum conditions, the medium was aspirated and replaced by new starvation medium with or without 10 ng/mL TGF-β1 (recombinant human, animal-free, PeproTech^®^, Thermo Scientific). Samples were collected for gene expression and protein analysis on day 2 and day 5 of treatment with TGF-β1. These timepoints were selected during optimization studies as treatment effects on α-SMA gene and protein levels were visible. Media for day-5 samples, including TGF-β1, were replaced in full on day 2.

#### 2.3.2. Quantitative Gene Expression Analysis (qPCR) and α-SMA Protein Expression

For gene expression analysis, scaffolds were washed three times in cold PBS, transferred to 0.5 mL TRIzol reagent (Invitrogen, Thermo Fisher) and stored at −80 °C until use (three replicates per condition, *n* = 3 donors). Before RNA isolation, scaffolds in TRIzol were thawed and manually crushed with a pipette tip followed by 30 min of incubation at room temperature to facilitate the release of genetic material. Next, the supernatants were transferred to new Eppendorf tubes and RNA was isolated using the RNeasy micro kit (Qiagen, Hilden, DE) according to the manufacturer’s instructions, including on-column DNA removal using RNAse-free DNAse as specified (Qiagen). RNA was eluted using RNAse-free water, and concentration and purity were measured using a NanoDrop 2000 spectrophotometer (Thermo Scientific). cDNA was prepared using the iScript^TM^ cDNA synthesis kit (Bio-Rad) according to the manufacturer’s instructions. Primer sets for the following targets were used: *GAPDH*, *B2M*, *YWHAZ*, *ACTA2*, *TGFB1*, and *COL1A1* (Supplementary Table S1, Biolegio, Nijmegen, NL). Quantitative PCR was performed using iQ^TM^ SYBR^®^ Green Sypermix (Bio-Rad, Hercules, CA, USA), with 2 ng cDNA and 15 pmol of each primer per reaction. Expression was measured using a CFX96 Touch Deep Well^TM^ Real-Time PCR detection system (Bio-Rad), set to 3 min of initial denaturation at 95 °C, followed by 40 cycles of denaturation (15 s at 95 °C), annealing/extension (30 s at 60 °C), and signal measurement, and ending with a melt curve analysis (10 s, 65–95 °C). Gene expression fold changes were calculated using the 2^−ΔΔCt^ approach relative to untreated cells at day 2 on 0% FGF2 collagen scaffolds in the absence of TGFβ1.

For protein expression analysis, scaffolds were stored immediately in reducing sample buffer at −20 °C (*n* = 3 donors). Samples were boiled 10 min before loading onto 15% polyacrylamide gels (Thermo Scientific). Protein separation, blotting, and blocking were performed as described in Section 2.2.4. GAPDH was labeled using rabbit-anti-GAPDH (clone 14C10, 1:1000, Cell Signaling Technology, Danvers, MA, USA) and goat-anti-rabbit IgG IRDye 680CW (1:15,000, LI-COR). α-SMA was labeled using mouse-anti-α-SMA (1:2000, Sigma-Aldrich) and goat-anti-mouse IgG IRDye 800CW (1:10,000, LI-COR). Antibodies were diluted in BSA-PBST and incubated on blots for 60 min. Blots were washed 3 × 5 min in PBST after each incubation. The blots were scanned and the signal was quantified as described (Section 2.2.4). The ratio of α-SMA over GAPDH was calculated and a fold change in α-SMA protein abundance was determined relative to untreated cells at day 2 on 0% collagen scaffolds.

#### 2.3.3. Data Analysis

Data visualization and the identification of significant differences were performed using GraphPad Prism (version 10.2.2). Differences in final heparin or OTR4120 content as a result of the scaffold production method was tested with a paired *t*-test including a two-stage step-up approach [30] with a False Discovery Rate of 1.00% (differences were deemed significant if q < 0.01). Three batches of scaffolds were tested (*n* = 3) and data are presented in grouped bar graphs displaying mean ± SD. The amount of FGF-2 captured by the scaffolds is presented as mean ± SD in bar graphs, and significant differences between conditions were tested using a one-way ANOVA with Tukey’s multiple comparisons test (α = 0.05); results were deemed significant if adjusted *p* < 0.05. Relative gene expression and relative protein expression are presented in grouped bar graphs as mean ± SD (*n* = 3). Differences in gene or protein expression levels between the scaffolds of one treatment condition (at one timepoint) were tested using a two-way ANOVA with Tukey’s multiple comparison test (α = 0.05), and results were deemed significant if adjusted *p* < 0.05.

## 3. Results

### 3.1. Production and Characterization of Scaffolds

Two methods of scaffold production were compared to identify the most appropriate method. In the “soaked” method, porous type I collagen scaffolds were incubated overnight in a solution containing heparin or OTR4120 to facilitate their distribution before crosslinking. In the “mixed” method, heparin or OTR4120 was added to the collagen fibrils during the swelling procedure. Mixing in 0.05% of heparin or OTR4120 impacted the swelling capacity of the collagen fibrils as some fibrils were not fully swollen and non-swollen fibrils were difficult to homogenize. This effect was not seen in the mixed conditions with 0.025–0.00625% of heparin or OTR4120. Visually, scaffolds from both methods were almost identical, with mixed scaffolds being thinner and more concave compared to their soaked counterparts (Supplementary Figure S1).

**Figure 1:**
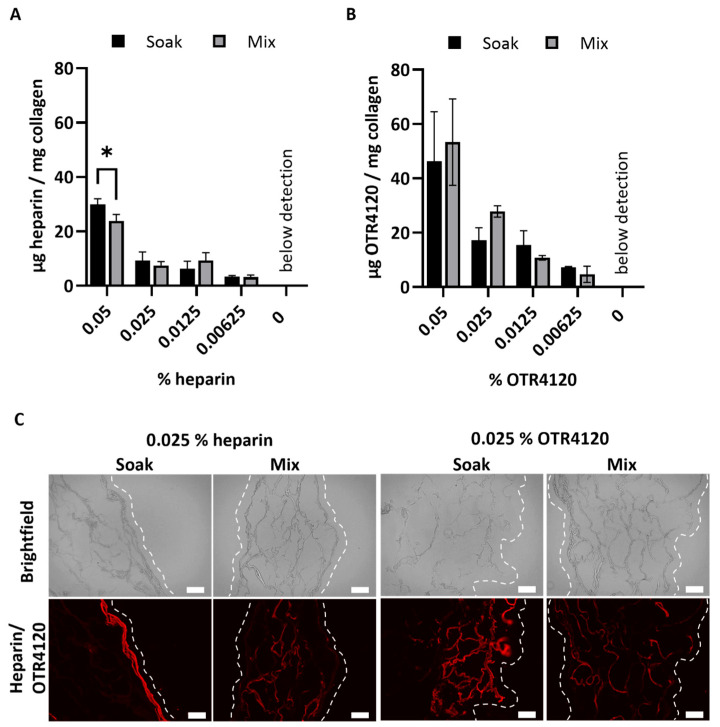
Effect of production method (“soak” versus “mix”) on the content and distribution of heparin or OTR4120 in collagen scaffolds after crosslinking. The final heparin (**A**) or OTR4120 (**B**) content of the scaffolds can be controlled by adjusting the % heparin or OTR4120 used during crosslinking. The heparin or OTR4120 content was not affected by the production method. Data are represented as mean ± SD (*n* = 3). Differences were tested with a paired *t*-test including a two-stage step-up approach with significance threshold of q < 0.01, * *p* = 0.0015. (**C**) Representative images for the localization of heparin or OTR4120 in cross-sections of collagen scaffolds with 0.025% heparin or OTR4120 using a single-chain antibody (HS4C3). Mixing in heparin or OTR4120 results in an even distribution throughout the scaffolds. Dashed line indicates outer edge of the scaffold. Scale bar is 100 μm.

After crosslinking, the heparin or OTR4120 content of the lyophilized scaffolds was quantified as μg/mg scaffold (mean ± SD) (Figure 1A,B). Scaffolds soaked in 0.05% heparin contained 30 ± 2 μg heparin/mg scaffold, which was significantly more compared to the mixed counterpart with 24 ± 2 μg heparin/mg scaffold (*p* = 0.0015). Heparin contents of 0.025% and lower were not affected by the production method and ranged from 3 to 9 μg heparin/mg scaffold for both methods. For OTR4120-functionalized scaffolds, the production method did not affect the final OTR4120 content. Scaffolds produced with 0.05% had the highest OTR4120 content, with 46 ± 18 μg OTR4120/mg scaffold for soaked scaffolds and 53 ± 16 μg OTR4120/mg scaffold for mixed scaffolds. The OTR4120 content decreased in a dose-dependent fashion (Figure 1B).

The distribution of heparin and OTR4120 throughout the scaffolds was visualized using immunofluorescence assays, which demonstrated noticeable differences between soaked and mixed conditions (Figure 1C). Soaking the scaffolds in heparin or OTR4120 resulted in the restriction of these compounds to the outer scaffold edges, whereas mixing in heparin or OTR4120 resulted in a more homogenous distribution throughout the scaffold. Additionally, the fluorescence intensity and overall coverage in the scaffolds diminished along with lower % of OTR4120 (Supplementary Figure S2). The mixing method using 0.025% heparin or OTR4120 was selected for further use as the resulting collagen suspensions were easy to homogenize and yielded scaffolds with an even distribution of heparin/OTR4120.

Scaffolds mixed with 0.025% heparin or OTR4120 were sterilized using γ-irradiation. Sterilization did not affect the heparin or OTR4120 content of the scaffolds (Supplementary Figure S3). Collagen-heparin scaffolds contained 20 ± 2 μg heparin/mg scaffold (mean ± SD) before sterilization compared to 19 ± 3 μg/mg after sterilization. Collagen–OTR4120 scaffolds contained 21 ± 1 μg OTR4120/mg scaffold before sterilization and 26 ± 2 μg/mg after sterilization. Possible breakdown of the collagen fibrils after γ-irradiation was assessed through SDS-PAGE (Supplementary Figure S4). Without crosslinking, the soluble fraction of type I collagen fibrils displayed the characteristic band pattern of α- and β-chains. Non-sterilized scaffolds did not display these bands, indicating that all collagen fibrils were crosslinked. The lanes obtained from γ-irradiated scaffolds displayed protein staining near/in the running front, indicating the presence of small protein fragments. This effect was present in both heparin and OTR4120 scaffolds, as well as in control scaffolds without any additives (0%). Overall, major signs of scaffold breakdown after sterilization were not observed.

### 3.2. Collagen–OTR4120 Scaffolds Capture FGF-2 Comparably to Collagen–Heparin

**Figure 2:**
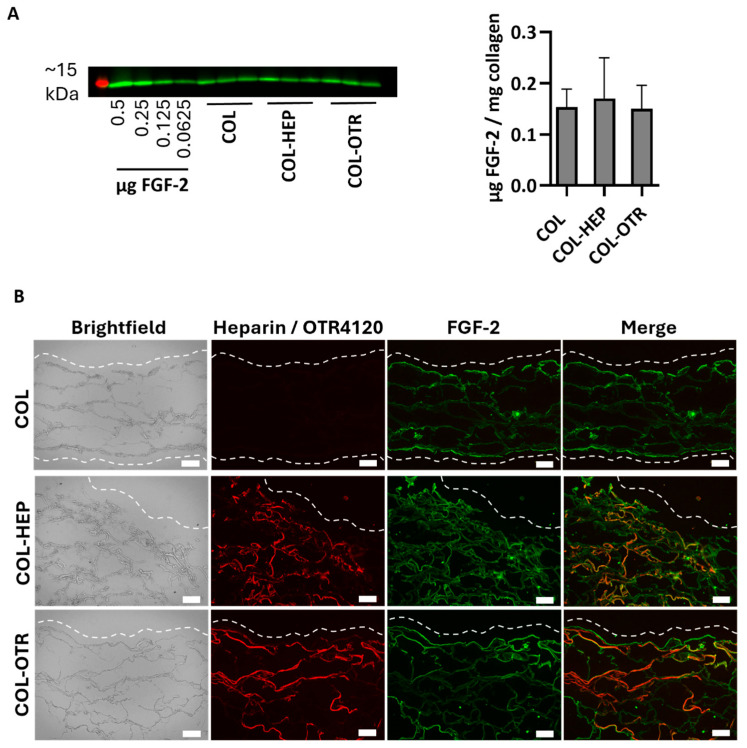
FGF-2 captured by γ-sterilized collagen scaffolds with mixed in 0.025% heparin (COL-HEP), 0.025% OTR4120 (COL-OTR), and no additives (0%: COL). (**A**) Image displays Western blot with the ~15 kDa protein marker in red and FGF-2 protein bands in green, with an FGF-2 standard series and the FGF-2 captured by COL, COL-HEP, and COL-OTR. Graph presents the quantified amount of FGF-2 captured, normalized to sample weight (mean ± SD, *n* = 3). All scaffold types captured similar amounts of FGF-2 (*p* > 0.05, one-way ANOVA with Tukey’s multiple comparison test and α = 0.05). (**B**) Representative images of indirect immune fluorescent assay on scaffold cross-sections, where brightfield shows the whole scaffold, HS4C3 (red) labels heparin or OTR4120, and FGF-2 is labeled in green. Merged images show co-localization of heparin/OTR4120 and FGF-2 in orange. COL-HEP and COL-OTR are evenly covered with heparin and OTR4120, respectively. The distribution of FGF-2 is similar across all three scaffolds, with co-localization with heparin (COL-HEP) and OTR4120 (COL-OTR) in various areas. Dashed line indicates the outer edge of the scaffold and scale bar is 100 μm.

As a heparan sulfate mimetic, OTR4120 should have the same ability as heparin to capture FGF-2, an anti-fibrotic heparin-binding growth factor. To this end, γ-sterilized collagen scaffolds mixed with 0.025% heparin (COL-HEP) or 0.025% OTR4120 (COL-OTR) or without an additional compound (0%: COL) were incubated with FGF-2 in PBS. First, the amount of FGF-2 captured by the scaffolds was assessed by Western blot (Figure 2A). All samples captured roughly the same amount of FGF-2, as COL scaffolds contained 0.15 ± 0.04 μg/mg, COL-HEP captured 0.17 ± 0.08 μg/mg, and COL-OTR captured 0.14 ± 0.04 μg/mg scaffold.

The distribution of heparin, OTR4120, and FGF-2 was visualized using immunofluorescence assays on cryosections (Figure 2B). Both COL-HEP and COL-OTR scaffolds displayed an even distribution of heparin or OTR4120 in the scaffold, while the sections of COL were devoid of staining. On the other hand, FGF-2 was present in all three scaffold types with a similar distribution pattern, where FGF-2 was concentrated at the scaffold edges with some of the signal penetrating further into the scaffolds. COL scaffolds displayed binding of FGF-2 on the (bare) collagen fibers. Merging of the heparin or OTR4120 and FGF-2 signal indicated that these compounds co-localized in various areas.

### 3.3. Collagen–OTR4120 Scaffolds Gradually Release FGF-2

**Figure 3:**
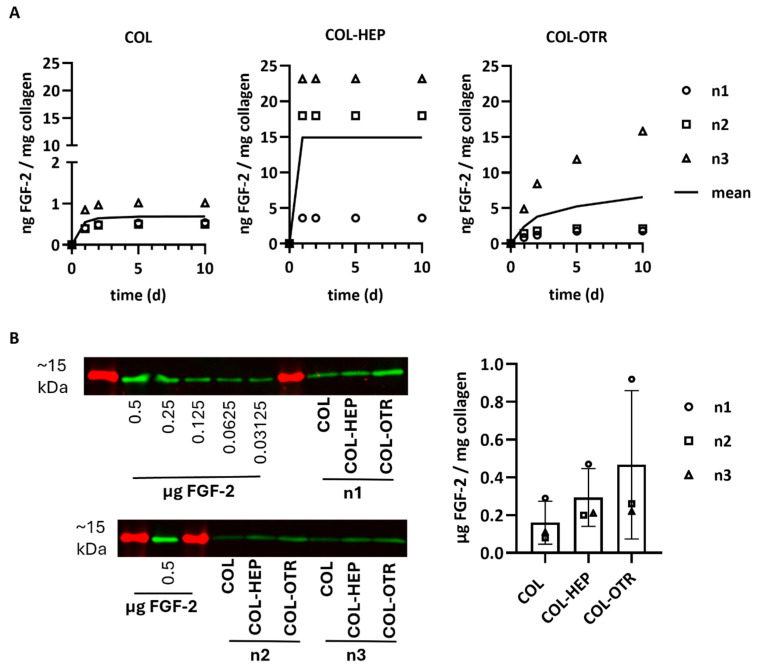
FGF-2 release profiles from γ-sterilized scaffolds with 0.025% heparin (COL-HEP) or 0.025% OTR4120 (COL-OTR) and without additives (0%: COL). (**A**) Cumulative release profiles of FGF-2 in PBS at 37 °C, where data points of three scaffold batches are presented along with a line representing the mean cumulative release (*n* = 3). COL released 10× less FGF-2 compared to the other scaffold type. COL-HEP burst-released FGF-2 within 1 day, whereas COL-OTR more gradually released FGF-2. (**B**) Quantification of FGF-2 remaining in the collagen scaffolds after 30 days of incubation in PBS. Western blot (left) shows the ~15 kDa protein marker (in red) and protein bands of FGF-2 (in green) for the standard curve and each batch of scaffolds (n1, n2, n3). The FGF-2 signal was quantified using the calibration curve and normalized to sample weight; the results are presented in the graph (mean ± SD, *n* = 3) and indicate that most of FGF-2 remains unreleased, with large differences between the scaffold batches (no statistical analysis was performed).

The release profiles of FGF-2 into PBS from scaffolds with/without heparin or OTR4120 was investigated via ELISA. Release was quantified at various timepoints as ng FGF-2 released/mg collagen (*n* = 3). The mean cumulative release profiles over 10 days are presented in Figure 3A. Considerable differences between scaffold batches were noticed, but the release profiles were similar within each scaffold type. Overall, COL released the least amount of FGF-2 with an average of 0.7 ng FGF-2/mg on day 10, of which the majority was released within the first 3 days. Both COL-HEP and COL-OTR released over 10-fold more FGF-2 compared to COL. COL-HEP scaffolds burst-released FGF-2 within the first day, with an average of 14.9 ng/mg, and no more FGF-2 was released after this timepoint. In contrast, the pattern of release seen with COL-OTR was different, displaying a more gradual increase in FGF-2 release over time, with a maximum of 6.6 ng/mg on day 10.

Any FGF-2 remaining in the scaffolds after 30 days of release was quantified via Western blotting and is presented as μg FGF-2/mg collagen (mean ± SD, Figure 3B). Most of the FGF-2 seemed to remain in the scaffolds even after 30 days in PBS. The variation between scaffold batches in the release profiles was also observed in the remaining amounts of FGF-2, resulting in large standard deviations. The smallest amount of FGF-2 remained on 0% scaffolds (0.16 ± 0.11 μg/mg), followed by collagen–heparin with 0.30 ± 0.15 μg/mg. OTR4120 scaffolds retained the highest amount at 0.47 ± 0.40 μg FGF-2/mg collagen.

### 3.4. Collagen–OTR4120(-FGF-2) Scaffolds Possess Inherent Anti-Fibrotic Properties In Vitro

**Figure 4:**
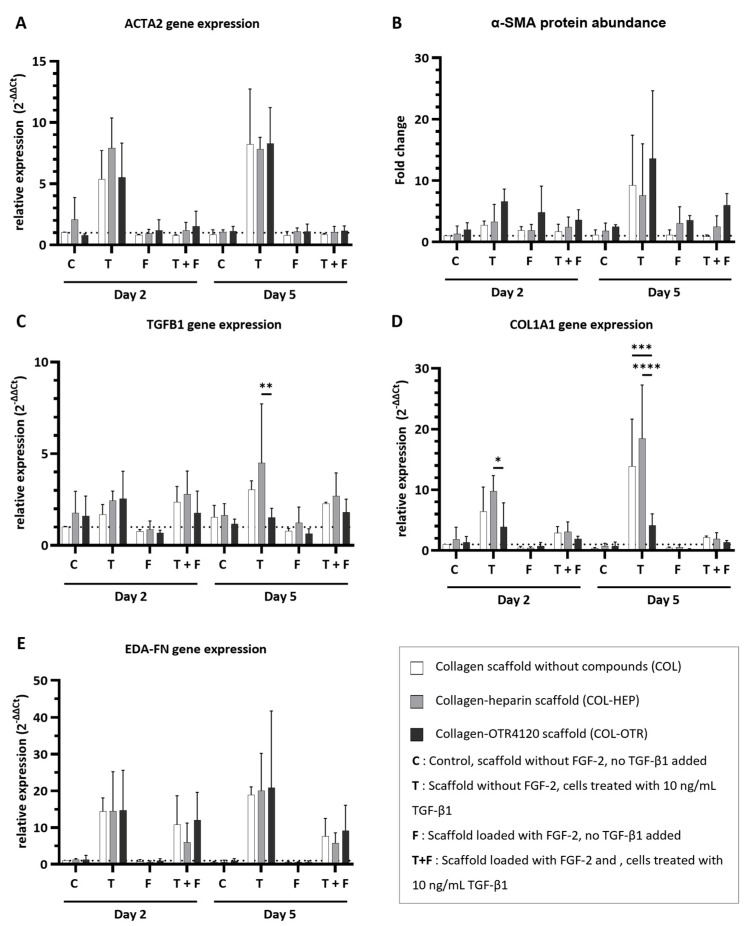
Effect of collagen–heparin (COL-HEP), collagen–OTR4120 (COL-OTR), and collagen-only (COL) scaffolds with/without FGF-2 on the expression of myofibroblast markers by primary human dermal fibroblasts. (**A**) α-smooth muscle actin gene expression levels (ACTA2). (**B**) α-smooth muscle actin protein abundance (α-SMA). (**C**–**E**) Gene expression levels of transforming growth factor β1 (TGFB1) (**C**); type I collagen A1 (COL1A1) (**D**); and fibronectin extra domain A (EDA-FN) (**E**). Overall, the presence of FGF-2 limited the upregulation of myofibroblast markers. OTR4120 had an inherent anti-fibrotic effect as HDFs on COL-OTR scaffolds expressed significantly less TGFB1 and COL1A1 in reaction to TGF-β1 treatment. Data are represented as mean ± SD and N = 3 (three separate donors). C = control scaffold without FGF-2 and no treatment with TGF-β1; T = scaffold without FGF-2 but cells were treated with 10 ng/mL TGF-β1; F = scaffold loaded with FGF-2 but no addition of TGF-β1; T + F = scaffold with FGF-2 and with addition of 10 ng/mL TGF-β1. Differences between COL, COL-HEP, and COL-OTR were tested with two-way ANOVA including Tukey’s multiple comparison test (α = 0.05); *: *p* = 0.036; **: *p* = 0.0019; ***: *p* = 0.0003; ****: *p* < 0.0001.

The ability of COL, COL-HEP, and COL-OTR scaffolds to reduce the fibrotic response was investigated in vitro (Figure 4). HDFs were cultured on top of scaffolds without FGF-2 or scaffolds pre-loaded with FGF-2 (the presence of FGF-2 is marked with ‘F’ in the plots). Cells were stimulated with TGF-β1 to induce myofibroblast differentiation (treatment with TGF-β1 is marked as ‘T’). Cells cultured on scaffolds without FGF-2 and in the absence of TGF-β1 were used as controls (marked ‘C’ in the plots). The expression levels of several myofibroblast markers were measured to determine the anti-fibrotic potential of the scaffolds.

The expression of α-smooth muscle actin, a key protein that is incorporated into the stress fibers of mature myofibroblasts, was determined at gene (*ACTA2*, Figure 4A) and protein (α-SMA, Figure 4B) levels. Determination of gene expression and protein abundance is an excellent indicator of the anti-fibrotic effects of the collagen scaffolds. Imaging of cells on scaffolds was attempted with immunofluorescence assays on cryosections during optimization studies of the in vitro cell culture model, but this method was not suitable for providing quantitative information about cell morphology (Supplementary Figure S5).

Treatment with TGF-β1 resulted in an upregulation of *ACTA2* expression across COL, COL-HEP, and COL-OTR, and an increase in α-SMA abundance was seen on day 5. The upregulation of *ACTA2* was prevented when scaffolds were enriched with FGF-2: *ACTA2* expression for all three scaffold types resembled that of untreated controls. The presence of FGF-2 on the scaffolds generally limited the increase in α-SMA abundance, but in COL-HEP and especially COL-OTR, the α-SMA abundance remained higher than untreated controls. No significant differences between scaffold types were found. Next, *TGFB1* expression was determined: this gene encodes the precursor of the TGF-β1 latency-associated peptide complex (Figure 4C). Treatment with TGF-β1 slightly increased *TGFB1* expression levels. Interestingly, on day 5, cells grown on COL-OTR expressed less *TGFB1* than COL-HEP (*p* = 0.0019). The added presence of FGF-2 did not have a major effect, as *TGFB1* expression levels remained above those of untreated controls. As myofibroblasts mainly produce type I A1 collagen (*COL1A1*), and its overabundance is a characteristic of fibrotic tissue, its expression levels were measured (Figure 4D). Exposure to TGF-β1 led to increased *COL1A1* expression across all scaffold types. Again, COL-OTR scaffolds had a different response to TGF-β1 treatment, with lower expression levels of COL1A1 than COL-HEP on day 2 (*p* = 0.0362) and compared to other scaffolds on day 5 (COL vs. COL-OTR, *p* = 0.0003; COL-HEP vs. COL-OTR, *p* < 0.0001). The presence of FGF-2 lowered *COL1A1* expression by 30–90%, and no differences between scaffold types were found. Lastly, extra domain A fibronectin (*EDA-FN*), a marker for both proto-myofibroblasts and mature myofibroblasts, was measured (Figure 4E). Treatment with TGF-β1 led to increases in *EDA-FN* expression across all three scaffolds. The presence of FGF-2 did not fully prevent this effect: while *EDA-FN* expression levels had halved at day 5, they remained higher than in untreated controls. No differences between COL, COL-HEP, and COL-OTR were found.

## 4. Discussion

A need remains for innovative biomaterials that can control the fibrotic response and stimulate skin regeneration. Here, we present a novel collagenous biomaterial that is based on an established approach where heparin/heparan sulfate is used to capture and present anti-fibrotic growth factors. OTR4120, a degradation-resistant heparan sulfate mimetic, was successfully linked to type I collagen scaffolds (COL-OTR). These COL-OTR scaffolds could be loaded with the anti-fibrotic heparin-binding growth factor FGF-2 and demonstrated anti-fibrotic properties in an in vitro model.

The COL-OTR scaffolds were compared with collagen–heparin (COL-HEP) and collagen-only (COL) scaffolds. First, the scaffold production method was optimized, and mixing in OTR4120 or heparin during swelling of the collagen fibrils resulted in an even distribution of these components throughout the scaffolds. The suspension was difficult to homogenize at the highest concentration of 0.05% OTR4120 or heparin. Collagen fibrils become positively charged during the swelling process at acidic pH, causing individual fibrils to repel each other and allowing fluid to invade [31,32]. The negatively charged sulfate groups on heparin and OTR4120 may counteract these positive charges, thus reducing the swelling capacity of the collagen at high concentrations of heparin or OTR4120 [33]. No problems were encountered during homogenization of the lower concentrations of heparin or OTR4120 (0.025–0%). Overall, scaffolds produced with 0.025% OTR4120 or heparin mixed into the collagen suspension were easy to homogenize. The resulting scaffolds contained similar amounts of OTR4120 or heparin, and the compounds were evenly distributed throughout the scaffolds.

The loading of scaffolds with an excess of FGF-2 showed that COL-OTR, COL-HEP, and COL captured similar amounts, ranging from 0.15 to 0.17 μg FGF-2/mg scaffold. The distribution of FGF-2 throughout these scaffolds was also similar and FGF-2 co-localized with OTR4120/heparin in various areas. It may be that the amount of OTR4120/heparin was not sufficient for providing a major increase in FGF-2 binding capacity compared to collagen-only scaffolds. A related study that reported on collagen scaffolds produced with ~50× more heparan sulfate yielded scaffolds containing 60 μg heparan sulfate/mg collagen that captured 1.3 μg rat recombinant FGF-2/mg collagen [21]. The same study also reported that collagen-only scaffolds captured 0.37 μg FGF-2/mg collagen, which is more than our results and may be explained by using an over two-fold higher concentration for loading FGF-2.

The cumulative release profiles of FGF-2 point at a functional difference between COL, COL-HEP, and COL-OTR scaffolds. Both COL and COL-HEP scaffolds demonstrated a burst release of FGF-2, with COL-HEP releasing over 10 times more FGF-2 compared to COL. An initial burst release of FGF-2 from collagen and collagen–heparan sulfate scaffolds has been reported [21]. In contrast, the release of FGF-2 from COL-OTR scaffolds occurred in a gradual fashion, suggesting that OTR4120 enables a slow release FGF-2, which could possibly apply to other heparin-binding growth factors as well. The mechanism behind this slower release is most likely due to a higher affinity of OTR4120 for FGF-2, either via heparin-binding sites or general electrostatic interaction [34,35]. Nevertheless, the gradual release underlines the benefits of OTR4120 for growth factor capture and presentation, as burst release remains an unwanted side effect in many biomaterials [36]. Moreover, slow release of FGF-2 is beneficial to the wound healing response as the targets (myofibroblasts) become active several days after wounding [7]. In our approach, a fundamental benefit of OTR4120 is its ability to bind and present FGF-2 whilst preserving its bioactivity by offering protection from degradation. We did not perform any experiments on the degradation resistance of our scaffolds, but these characteristics of RGTA^®^ have been documented extensively [24].

To investigate the scaffold anti-fibrotic properties, an in vitro study was performed with myofibroblasts. Fibrosis was mimicked by TGF-β1-stimulated primary human dermal fibroblasts seeded on the scaffolds. COL-OTR, COL-HEP, and COL scaffolds loaded with FGF-2 all prevented the upregulation of myofibroblast-related markers *ACTA2*, *TGFB1*, *COL1A1,* and *EDA*-*FN*. No differences in anti-fibrotic potential between the FGF-2-loaded scaffolds were observed, which may be explained by the equal amounts of FGF-2 present in all three scaffolds. More remarkable were the inherent anti-fibrotic properties of COL-OTR scaffolds (in absence of FGF-2). When stimulated with TGF-β1, fibroblasts seeded on COL-OTR scaffolds expressed significantly less *TGFB1* and *COL1A1* compared to COL-HEP and COL scaffolds. This indicates that the interplay between OTR4120 and TGF-β1 is anti-fibrotic in the context of our in vitro model. TGF-β1 is a member of the heparin-binding protein family. The binding affinity of TGF-β1 to heparan sulfate/heparin is partially affected by the sulfation pattern of the glycosaminoglycan chains, resulting in heterogenous affinity levels [37]. There is no consensus on the effects of TGF-β1 signaling when this cytokine is sequestered by heparan sulfates/heparin [38]. Our results suggest that OTR4120, having a high affinity for TGF-β1, diminishes the fibrotic response by decreasing TGF-β1 bioavailability. OTR4120’s affinity for TGF-β1 can be investigated through competitive binding assays [39] or via a Western blot approach as demonstrated in this work. The reduction in *COL1A1* gene expression is especially relevant as fibrosis is hallmarked by an overabundance of type I collagen, and reducing (not blocking) its deposition had positive effects on wound healing [40]. This inhibitory effect of RGTA^®^ on collagen production has been reported in a study where a combination of RG-1503 (the previous name of OTR4120) and TGF-β1 reduced type I collagen production by smooth muscle cells compared to TGF-β1-treated controls [41]. Taken together, the data demonstrate that OTR4120 partially diminishes the TGF-β1-mediated fibrotic response. It is worthwhile to further investigate the interaction between TGF-β1 and OTR4120 in future studies.

Future research should also use models that more closely recapitulate wound healing to address the anti-fibrotic nature of COL-OTR alone, as well as the added benefit of FGF-2. Wound healing is a notoriously complex process that is difficult to model [42]. The in vitro fibrotic model we used provides valuable information on the primary response of (myo)fibroblasts and the results strongly indicate an anti-fibrotic effect of COL-OTR(-FGF-2) scaffolds. Alternatively, the use of eschar fibroblasts might be useful to mimic burn wound conditions (Kutluoglu et al., in revision) [43]. Animal models, for example mouse or rat full-thickness skin wound models, are often used and provide information on biocompatibility and regenerative potential in a complex environment [44]. Nevertheless, alternatives to animal testing need to be seriously considered, especially given the advancements in complex in vitro skin models over recent years [45].

One point that needs addressing is the difference between various scaffold batches. In particular, the variations in FGF-2 capture and release are notable. The reasons behind these discrepancies are difficult to pinpoint. One explanation can be found in the distribution of heparin and OTR4120 throughout the scaffolds. Although mixing in these components markedly improved their distribution, the coverage was not entirely homogenous. When heparin and OTR4120 were visualized in immunofluorescence assays, regions within one scaffold showed varying levels of staining intensity. These effects are visible in the images of COL-OTR scaffolds presented in Supplementary Figure S2. Samples that were taken for analysis may thus have been subject to both inter- and intra-scaffold differences. Future studies should aim to improve this aspect of the COL-OTR scaffolds as product heterogeneity is a definite limitation on the path towards clinical application [46]. Additional considerations for future research are the scaffold mechanical characteristics and pore architecture, which were outside the scope of this work.

The (original) regenerative potential of OTR4120 lies in its ability to stabilize the wound matrix and restore growth factor signaling [24]. Our approach has demonstrated the ability of OTR4120 to gradually release FGF-2, and its inherent anti-fibrotic effects strongly indicate COL-OTR scaffolds could outperform the heparin-based approaches. Additionally, this work presents the first application where OTR4120 is covalently coupled to a biomaterial, and we demonstrated that the growth factor-protecting and anti-fibrotic properties are maintained. This presents new avenues for the application of RGTA^®^ in addition to the conventional approaches (such as creams, injections, or dermal application).

In conclusion, we presented a collagen-based biomaterial that harnessed the regenerative power of degradation-resistant heparan sulfate mimetics such as RGTA^®^. We were able to covalently crosslink the RGTA^®^ OTR4120 to porous scaffolds made from type I collagen. These novel collagen–OTR4120 scaffolds captured the anti-fibrotic growth factor FGF-2 and, in vitro, both collagen–OTR4120 and collagen–OTR4120-FGF2 scaffolds reduced myofibroblast differentiation. Collagen–OTR4120 scaffolds are a promising material for reducing skin fibrosis and promoting skin regeneration.

## Data Availability

Research data associated with this publication will be made available at the Radboud data repository under a CC-BY-NC 4.0 license at DOI 10.34973/ydab-rd48 upon publication of the manuscript.

## Supplementary Materials

The following supporting information can be downloaded at https://www.mdpi.com/article/10.3390/jfb16020051/s1. Table S1: Primer sequences for quantitative polymerase chain reaction. All primers were validated before use: validation criteria were met if r^2^ > 0.98 and efficiency was 95–105 %. Figure S1: Collagen scaffolds after crosslinking and lyophilization, representative scaffolds. Soak: scaffolds were soaked in a solution containing heparin or OTR4120 followed by crosslinking; Mix: heparin or OTR4120 was added to type I collagen fibrils during swelling, the suspension homogenized, and porous scaffolds created that were subsequently crosslinked. Heparin and OTR4120 scaffolds looked visually identical. Mixed scaffolds were overall thinner, and more concave compared to soaked scaffolds. Figure S2: Immune fluorescence assay on cross sections of mixed collagen scaffolds with various concentrations of OTR4120 (0.05% = 0.5 mg OTR4120/mL collagen suspension). Brightfield images show all fibers in the scaffold. OTR4120 was labeled in red using the heparin/OTR4120-specific single chain antibody HS4C3. In each condition OTR4120 is distributed throughout the entire scaffold. As % decreases the coverage becomes more heterogenous, the staining intensity of OTR4120 is highest in 0.05% scaffolds and lowest in 0.00625% scaffolds. Representative images (*n* = 2), dashed line marks the outer edge of the scaffold. Scale bar is 100 μm. Figure S3: Effect of γ-irradiation on scaffolds with 0.025% mixed in heparin or OTR4120. After γ-irradiation, there was no change in the heparin or OTR4120 content of the scaffolds (paired *t*-test including two-stage step-up approach 2 and False Discovery Rate = 1.00%). Data are represented as mean ± SD (*n* = 3). Figure S4: Effect of sterilization via γ-irradiation on collagen scaffolds visualized by SDS-PAGE. Proteins are stained blue using Coomassie brilliant blue. Each lane represents a separate batch (*n* = 3). Control: purified, uncrosslinked type I collagen fibrils, displaying the characteristic band pattern of α1, α2, and β chains. Sterilized, crosslinked scaffolds do not display these chains, which is indicative of crosslinked collagen fibrils. The lanes from γ-irradiated scaffolds display small protein fragments (indicated by blue staining near the running front) but signs of scaffold breakdown were not observed. Figure S5: Morphology of human fetal lung fibroblasts (HFL1) cells cultured on collagen scaffolds. HFL1 cells were seeded on collagen-only (0%) scaffolds and cultured for 5 days in culture medium (Ham’s F12K medium (Gibco, Thermo Scientific) supplemented with penicillin-streptomycin and 0.5% fetal bovine serum) with or without 10 ng/mL transforming growth factor β1 (TGF-β1). Cryosections were prepared as described in the materials and methods Section 2.2.3. Immunofluorescence assays were performed as described in Section 2.2.3. F-actin was labelled with Alexa FluorTM 488 phalloidin (1:400, Invitrogen, Thermo Scientific). α-smooth muscle actin fibers (αSMA) were labeled with mouse-anti-α-SMA (1:2000, Sigma-Aldrich) and goat-anti-mouse Alexa FluorTM 594 (1:500, Invitrogen, Thermo Scientific). Nuclei were stained with DAPI (4′,6-diamidino-2-phenylindole). Merged image shows the overlay of DAPI, F-actin and αSMA signal. Brightfield image shows the scaffold morphology with the dashed lines marking the outer scaffold edge and ‘*’ indicating the inside of the scaffold. Data show that cells remain near the scaffold edge and no additional information about cell morphology can be gained. Scale bar is 100 μm and data are from optimization studies of the cell culture model (*n* = 2, representative images from n1 shown). References [30,47] are cited in the Supplementary Materials.
