# Supplementary material for: Next-Generation Biomaterials for Wound Healing: Development and Evaluation of Collagen Scaffolds Functionalized with a Heparan Sulfate Mimic and Fibroblast Growth Factor 2
Source: J Funct Biomater. 2025 Feb 7;16(2):51. doi: 10.3390/jfb16020051 (PMC11856099; doi:10.3390/jfb16020051)
Supplement: Supplementary file 1 [file jfb-16-00051-s001.zip › jfb-3411519-supplementary.pdf]

Supplementary Table S1: Primer sequences for quantitative polymerase chain reaction. All primers were validated before use: validation criteria were met if $r^2 > 0.98$ and efficiency was 95-105 %.

Symbol	Gene name	Primer sequence
GAPDH *	Glyceraldehyde 3-phosphate dehydrogenase	5'-TGGGTGTGAACCATGAGAAG-3'<br>3'-AGTTGTCATGGATGACCTTGG-5'
B2M *	Beta-2-microglobulin	5'-ATGAGTATGCCTGCCGTGTGA-3'<br>3'-GGCATCTTCAAACCTCCATG-5'
YWHAZ *	Tyrosine 3-Monooxygenase/Tryptophan 5-Monooxygenase Activation Protein Zeta	5'-CATCTTGGAGGGTCGTCTCA-3'<br>3'-ACTTTGCTCTCTGCTTGTGAA-5'
ACTA2 [1]	Actin alpha 2 , smooth muscle	5'-CCGACCGAATGCAGAAGGA-3'<br>3'-ACAGAGTATTTGCGCTCCGAA-5'
TGFB1	Transforming growth factor beta 1	5'-CGCGTGCTAATGGTGGAAA-3'<br>3'-TGTGTGTACTCTGCTTGAACTTGTCA-5'
COL1A1	Type I collagen, alpha 1 chain	5'-GAGAGCATGACCGATGGATT-3'<br>3'-CGCTGTTCTTGCAGTGGTAG-5'

** reference genes*

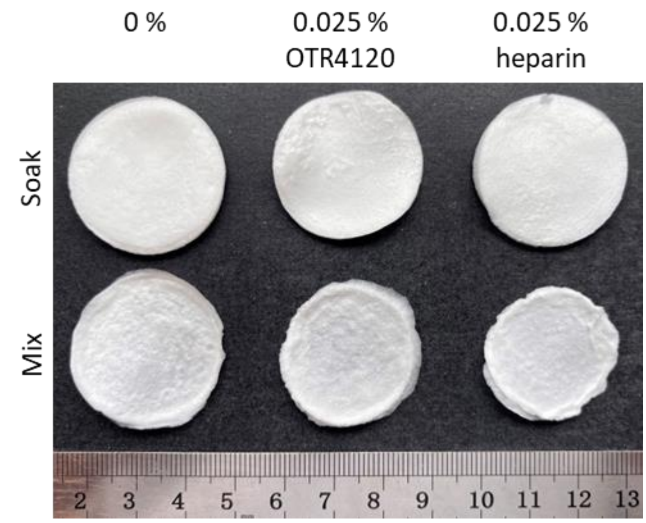

Supplementary Figure S1: Collagen scaffolds after crosslinking and lyophilization, representative scaffolds. Soak: scaffolds were soaked in a solution containing heparin or OTR4120 followed by crosslinking; Mix: heparin or OTR4120 was added to type I collagen fibrils during swelling, the suspension homogenized, and porous scaffolds created that were subsequently crosslinked. Heparin and OTR4120 scaffolds looked visually identical. Mixed scaffolds were overall thinner, and more concave compared to soaked scaffolds.

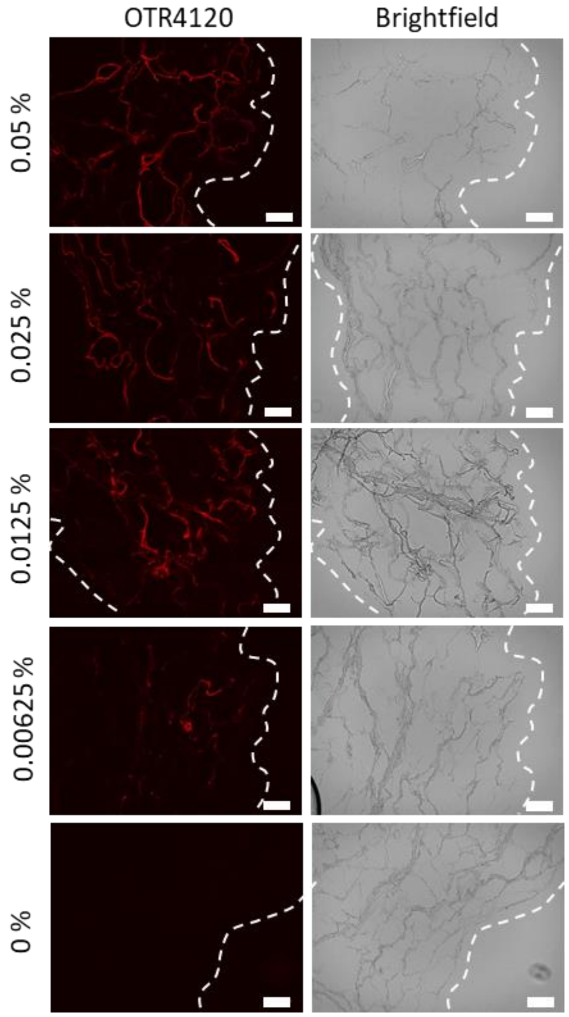

Supplementary Figure S2: Immune fluorescence assay on cross sections of mixed collagen scaffolds with various concentrations of OTR4120 (0.05 % = 0.5 mg OTR4120 / mL collagen suspension). Brightfield images show all fibers in the scaffold. OTR4120 was labeled in red using the heparin/OTR4120-specific single chain antibody HS4C3. In each condition OTR4120 is distributed throughout the entire scaffold. As % decreases the coverage becomes more heterogenous, the staining intensity of OTR4120 is highest in 0.05 % scaffolds and lowest in 0.00625 % scaffolds. Representative images (n = 2), dashed line marks the outer edge of the scaffold. Scale bar is 100 µm.

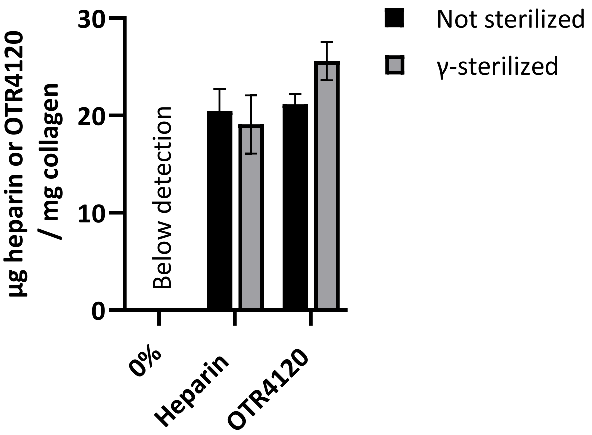

Supplementary Figure S3: Effect of γ-irradiation on scaffolds with 0.025 % mixed in heparin or OTR4120. After γ-irradiation, there was no change in the heparin or OTR4120 content of the scaffolds (paired t-test including two-stage step-up approach [2] and False Discovery Rate = 1.00 %). Data are represented as mean ± SD (n = 3).

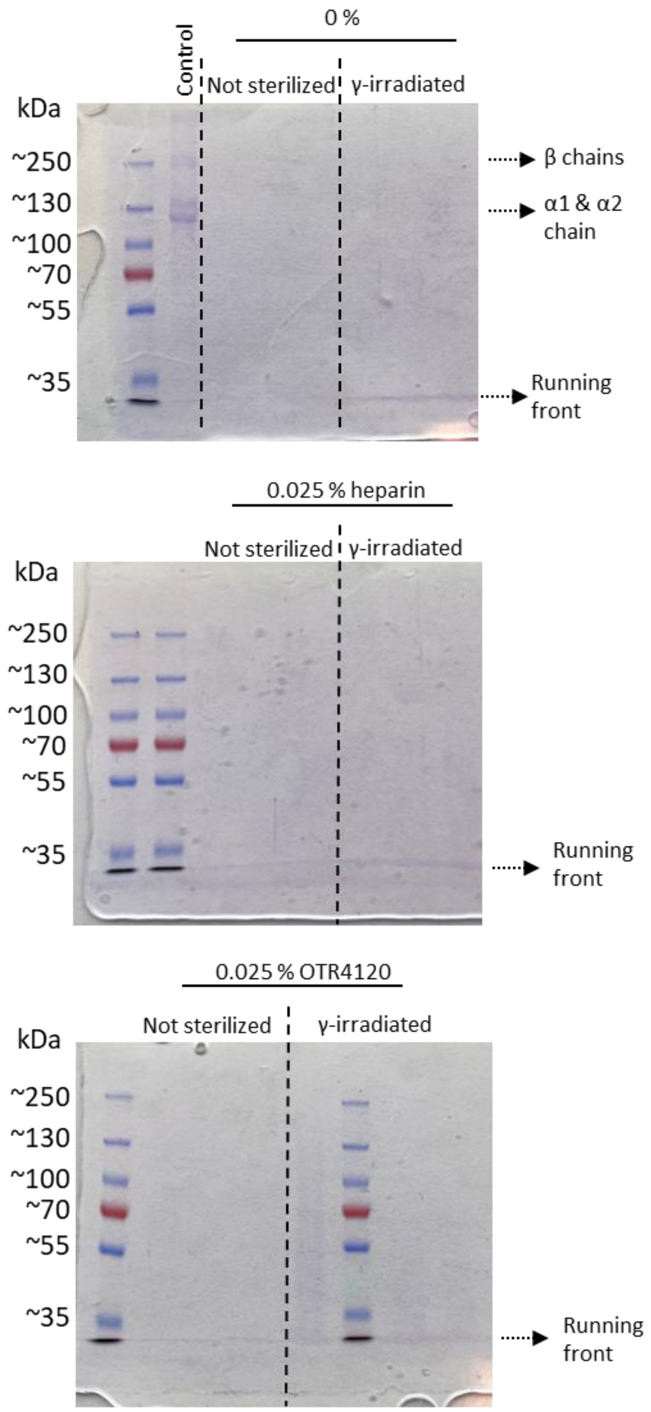

Supplementary Figure S4: Effect of sterilization via γ-irradiation on collagen scaffolds visualized by SDS-PAGE. Proteins are stained blue using Coomassie brilliant blue. Each lane represents a separate batch (n = 3). Control: purified, uncrosslinked type I collagen fibrils, displaying the characteristic band pattern of α1, α2, and β chains. Sterilized, crosslinked scaffolds do not display these chains, which is indicative of crosslinked collagen fibrils. The lanes from γ-irradiated scaffolds display small protein fragments (indicated by blue staining near the running front) but signs of scaffold breakdown were not observed.

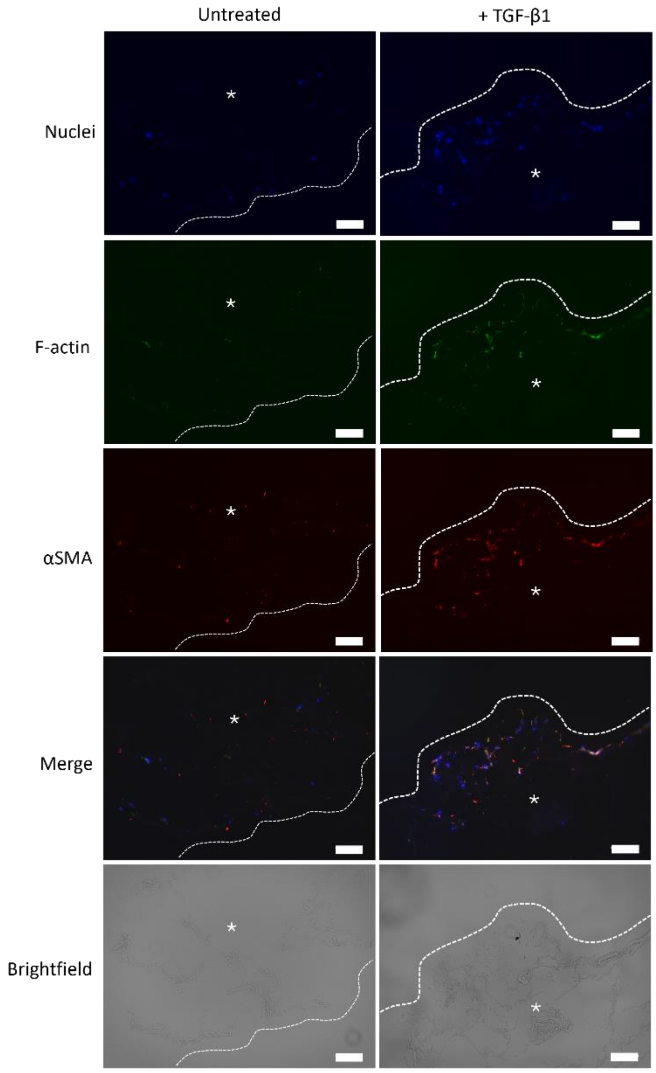

Supplementary Figure S5: Morphology of human fetal lung fibroblasts (HFL1) cells cultured on collagen scaffolds. HFL1 cells were seeded on collagen-only (0%) scaffolds and cultured for 5 days in culture medium (Ham's F12K medium (Gibco, Thermo Scientific) supplemented with penicillin-streptomycin and 0.5% fetal bovine serum) with or without 10 ng/mL transforming growth factor β1 (TGF-β1). Cryosections were prepared as described in the materials and methods section 2.2.3. Immunofluorescence assays were performed as described in section 2.2.3. F-actin was labelled with Alexa Fluor™ 488 phalloidin (1:400, Invitrogen, Thermo Scientific). α-smooth muscle actin fibers (αSMA) were labeled with mouse-anti-α-SMA (1:2000, Sigma-Aldrich) and goat-anti-mouse Alexa Fluor™ 594 (1:500, Invitrogen, Thermo Scientific). Nuclei were stained with DAPI (4',6-diamidino-2-phenylindole). Merged image shows the overlay of DAPI, F-actin and αSMA signal. Brightfield image shows the scaffold morphology with the dashed lines marking the outer scaffold edge and '*' indicating the inside of the scaffold. Data show that cells remain near the scaffold edge and no additional information about cell morphology can be gained. Scale bar is 100 µm and data are from optimization studies of the cell culture model (n=2, representative images from n1 shown).

References

1. Goldberg MT, Han YP, Yan C, Shaw MC, Garner WL. TNF-alpha suppresses alpha-smooth muscle actin expression in human dermal fibroblasts: an implication for abnormal wound healing. J Invest Dermatol. 2007;127(11):2645-55. Doi: 10.1038/sj.jid.5700890.
2. Benjamini, Y., A. Krieger, and D. Yekutieli, Adaptive linear step-up procedures that control the false discovery rate. Biometrika, 2006;93(3):491-507. Doi: 10.1093/biomet/93.3.491.
